# The Volebny Kompas Datasets on Slovak Voter and Party Positions

**DOI:** 10.1038/s41597-024-03777-0

**Published:** 2024-09-04

**Authors:** Jozef Michal Mintal, Kamila Borseková, Lorenzo Cicchi, Vladimír Müller, Róbert Vancel, Patrícia Šimková, Kevin Deegan-Krause

**Affiliations:** 1 Matej Bel University, Banská Bystrica, Slovakia; 2 European University Institute, San Domenico di Fiesole, Italy; 3 Wayne State University, MI Detroit, USA

**Keywords:** Interdisciplinary studies, Politics, Society

## Abstract

Three decades after the fall of socialist regimes in Central Europe, the successes of democratic transition face threats from broader trends toward skepticism, disengagement and distrust across Europe, though perhaps more intensively in the East. This backdrop, especially evident in Slovakia, underscores the need for comprehensive data on voter and political party positions during elections, a key opportunity for democratic renewal. Our article introduces two datasets from the Volebný Kompas project: the first, is a collection of 134,699 voter responses on 39 key issues, obtained through the Volebný Kompas voting advisory application for the 2023 snap Slovak Parliamentary Elections. The second dataset details the positions of 11 political parties on the same issues. Together, they provide a comprehensive view of Slovakia’s political landscape before a pivotal election which resulted in a dramatic change in government composition. As freely available and validated resources, these datasets can serve as an essential tool for understanding and navigating the evolving landscape of post-socialist democracies.

## Background & Summary

The fall of socialist regimes in Central Europe signaled a shift towards democracy, initially met with optimism for political freedom akin to Western democracies^1^. Three decades later, it is evident that the transition in many post-socialist countries remains incomplete^2^. These nations not only continue to lag behind in the level of infrastructure and quality of institutions, but also face a complex reality characterized by a growing scepticism towards liberal democracy, increased political disengagement, and a deep-seated distrust in political institutions — a situation that sharply contrasts with the initial euphoria of the post-socialist era^1,3–5^. These tensions are especially pronounced during election seasons. On one hand, elections offer an opportunity for democratic renewal, enabling citizens to articulate their preferences and hold institutions to account, thereby reaffirming their commitment to democratic ideals and addressing the root causes of cynicism^6^. On the other hand, it is common—perhaps increasingly common—for politicians to use elections to exploit fear and other negative emotions to exacerbate democratic discontent. In certain circumstances, especially when sentiments of discontent and fear are distributed asymmetrically across political parties, elections may bring to power coalitions that may pursue their goals in ways that are at odds with democratic norms or may even seek goals that are explicitly antidemocratic^7–9^.

While these developments are often most closely associated with post-socialist countries, they have also begun to appear in more established democracies such as the United States, the United Kingdom, France and elsewhere in Western Europe^10,11^. Understanding the causes of these developments is crucial for understanding the broader threat of democratic backsliding, and pivotal elections such as Slovakia’s 2023 vote offer useful examples. Until recently, however, data capturing the social and political positions of voters and political parties in such elections have been scarce or incomplete in coverage. This gap is particularly pronounced in the context of national elections, which are rendered impractical for large cross-national surveys due to the high heterogeneity and volatility of key social and political issues in elections that vary across time and geographical boundaries^12–15^.

In this article, we present the Volebný Kompas (eng. *Election compass)* data, composed of two different datasets: a comprehensive and freely available collection of over 134,000 voter responses, gathered during the 26 days leading up to and including the day of the 2023 snap Slovak Parliamentary Elections, along with a detailed record of 429 policy positions from 11 political parties. These datasets offer an in-depth picture of the electorate’s and parties’ positions on 39 crucial political, economic, and societal issues, detailed in Supplementary Table 1. The topics addressed in these datasets encompass a wide array of subjects, including but not limited to Foreign Policy and Defense, Criminal Justice, Taxation and Economic Policy, Environmental and Energy Policy, Civil Rights, and various Social Issues. The datasets’ extensive coverage of these diverse issues, paired with the users’ demographic data, creates an indispensable asset for a variety of scholarly disciplines. Their utility extends to facilitating the identification of distinct voter segments, examining the nuances of political polarization and ideological fragmentation, exploring the dynamics of ethnic politics, analyzing party dynamics, as well as discovering specific anomalies in behaviors across a range of issues. This not only allows for a more detailed analysis within the Slovak electoral context but also holds significance for broader research into contemporary European political and social developments.

The data originate from the Volebný Kompas voting advice application (VAA), which has emerged as the most prominent — and also only academic — VAA employed during the 2023 Slovak elections. Originally designed to guide voters toward their ideologically closest parties or candidates based on their policy preferences, VAAs have now taken on a dual function. They serve as rich repositories of data on public opinion and political behavior, shedding light on broader ideological dimensions^16^, stances on issues like same-sex marriage^17^, the impact of economic conditions on attitudes towards refugees^18^, or vote switching^19^. Their growing ubiquity has led scholars and political analysts to value VAAs both as tools for voter assistance and as reliable sources of data^34^ for understanding the nuances of modern political dynamics.

In this context, Slovakia, situated in Central Europe and characterized by one of the highest levels of distrust in governing institutions, anti-Western sentiments, and one of the lowest levels of support for liberal democracy in the region^4^, represents a critical ground for studying both political dynamics and the wider attitudinal shifts in the region. With its snap parliamentary elections held on September 30, 2023, Slovakia has become a focal point for political analysis^20–22^. The Slovak political landscape has undergone dramatic shifts since 2020, with the ruling parties’ support plummeting from a constitutional majority to 17%^20^. The political comeback of Robert Fico’s Smer party, once seemingly relegated to the political sidelines^23^, could as some worry signal a shift in Slovakia’s foreign policy, particularly regarding its position on Ukraine, which may affect the unity of the EU in their stance against Russia^21,22^. Domestically, there are growing concerns about the potential weakening of democratic institutions, as well as the erosion of human rights, social justice, and the rule of law^24^. These concerns underscore the broader challenges faced by many contemporary democracies in Central and Eastern Europe, marked, among others, by the rise of populist politics and the influence of foreign actors such as Russia^25^. With all these factors converging in Slovakia’s 2023 election, it serves as a microcosm of the broader political trends shaping the region.

**Fig. 1:**
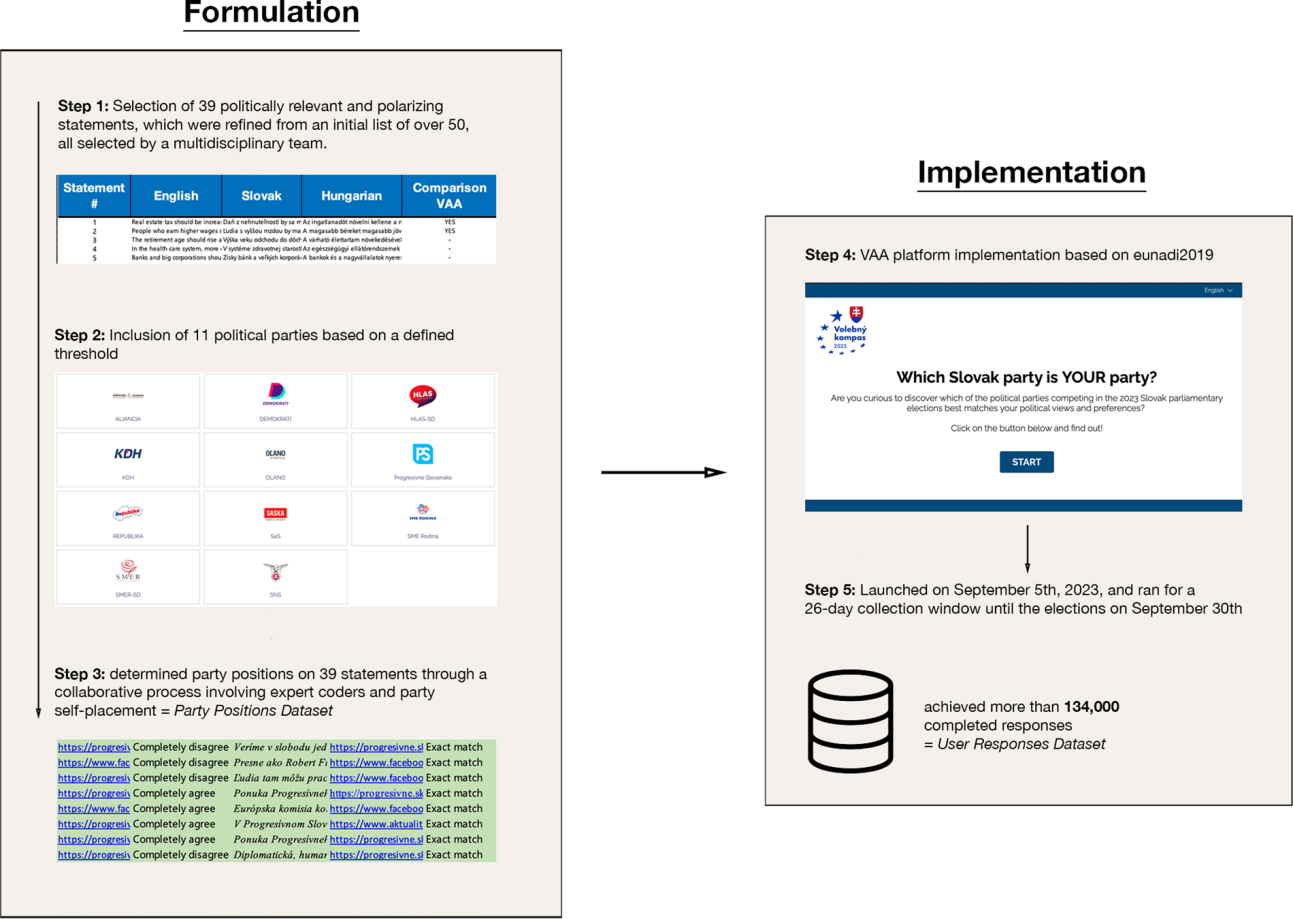
Overview of the Volebný Kompas workflow.

In the following sections of this paper, we provide an in-depth account of our data collection, processing, and validation methods. The creation process for the Volebný Kompas datasets is illustrated in Fig. 1, with subsequent sections detailing each step involved.

## Methods

### Ethics

The human subjects research in this article was reviewed and approved by the Ethics Committee at Matej Bel University (approval number 467/2023) as part of the Horizon Europe BRRIDGE project. Users provided consent for their data to be used for academic research and published in a pseudo-anonymized form.

### Statement selection

The foundation of our data collection project is rooted in the political statements upon which parties are positioned. The process of selecting these statements is paramount and serves as a cornerstone when developing a Voting Advice Application (VAA)^26^. The primary criteria for our statement selection were twofold:

- **Polarization**: The statements should be polarizing, meaning there should be parties that concur with the statement and parties that dissent. This is essential for distinguishing party stances for VAA party recommendations^26^.
- **Salience**: The statements should be salient, addressing political issues that hold significance in the respective electoral context, thereby ensuring that the VAA remains pertinent to the current political discourse^27^.

To draft the statements for Volebný Kompas, we assembled a multidisciplinary team comprising both Slovak and international scholars. This collaborative effort initially yielded a list of over 50 statements. Following intensive intra-group deliberations, the list was refined to a definitive set of 39 statements (see Supplementary Table 1). This number is consistent with the range typically employed in VAAs in other countries^28,29^. The statement selection methodology, including the final selection, was informed by the euandi methodology^30^. To determine the most salient issues, we relied on opinion polls, available party manifestos, and insights from team member expertise. We also considered longitudinal and comparative relevance with statements used in other VAAs, as well as potential data relevance to a diverse spectrum of disciplines. Several statements integrated into our definitive list have their origins or were directly employed in prior VAAs. We drew from previous iterations of transnational VAAs, including the EU Profiler for the European Parliament (EP) elections in 2009, euandi for the EP elections in 2014 and 2019, as well as national VAAs like Valijakompass 2023 in Estonia. Utilizing this methodology not only enhances the empirical robustness and validity of our dataset but also facilitates its comparison with existing datasets, offering interesting comparative insights across EU countries. This multi-faceted approach to statement selection ensured that the chosen statements were not only polarizing and salient to the elections—thereby enhancing the precision of the VAA recommendations from the outset—but also relevant to a diverse set of research fields and research questions. In designing and wording our statements, we drew upon best practices from VAA and survey research literature as guiding principles^31,32^. Given the substantial presence of the ethnic Hungarian minority in southern Slovakia, all finalized statements, originally formulated in both English and Slovak, were subsequently also translated into Hungarian by a professional translation agency.

### Party selection and coding

In the context of the 2023 snap general elections in Slovakia, where a total of 25 parties contested, determining which parties to include in Volebný Kompas was of paramount importance. Our selection criteria were grounded in empirical data spanning the six months leading up to July 2023. During this period, any party that achieved a minimum of 3% in any public poll conducted by an agency that is a member of the Slovak Association of Market Research Agencies (SAVA) was automatically incorporated into our VAA^33^. Our reliance on SAVA membership agencies further ensures the credibility and reliability of the data, given the association’s adherence to stringent research standards. It’s noteworthy that this 3% threshold is below the 5% required for a party to gain entry into the Slovak assembly. The rationale behind adopting a more inclusive benchmark is twofold. Firstly, it ensures a comprehensive analysis by including parties that, while not meeting the assembly entry threshold, still have a significant presence in the political landscape. Secondly, this 3% aligns with Slovak legislation, which mandates specific financial contributions for parties that achieve such a percentage in national elections^34^. Out of 25 political parties, 11 met the inclusion criteria and were incorporated into our VAA. The inclusion closely corresponded to election results, correctly anticipating the top 11 vote recipients. All of the 11 selected parties received at least 2% of the vote while no excluded party reached the 1%. Of the 13 parties that gained more than 1% of the vote in Slovakia’s 2020 election, eight were directly included in the list of 11, three merged with or campaigned on lists of other parties on the list of 11, one merged with a party that did not poll above 3% during polling (and received 0.3% of the votes) and one did not campaign for election^20^.

The challenge of accurately determining party positions across our set of 39 statements was a foundational aspect of our study. Our methodological approach was based on the euandi methodology, which itself is rooted in the iterative technique pioneered by the Dutch Kieskompas in 2006^28,35^. This methodology diverges from traditional expert survey methodologies, such as the Chapel Hill Expert Survey, which predominantly rely on the aggregation of individual expert placements. The reliability and validity of the euandi method vis-à-vis other data sources have been previously established, reinforcing the credibility of VAA-based methods in estimating the policy positions of European political parties^36^.

Our coding process of party positions was spearheaded by two independent expert coders. Parties were positioned on a Likert scale that spanned from “Completely Disagree” to “Completely Agree”, with intermediary categories of “Tend to Disagree”, “Neutral”, and “Tend to Agree”. To account for instances where parties remained non-committal, a “No Opinion” category was also included. To enhance the transparency and credibility of our coding process, coders were required to specify their sources along with a text snippet that supported their placements. Following the euandi methodology^29,30^, a hierarchy of sources was established, with the 2023 parliamentary election manifestos serving as the primary reference. In cases where this primary source was insufficient, coders sequentially consulted the party programme, other official party documents, public communications from party leaders, previous election manifestos, and finally other pertinent sources. To asses inter-coder agreement for our party stance coding, we employed Weighted Cohen’s Kappa^37^, which provided a value of κ = 0.84, indicating a high level of consistency among coders.

In tandem with expert coding, we initiated engagement with all 11 parties that satisfied our inclusion criteria through a self-placement questionnaire utilizing the same Likert scale as the one used by our two independent coders. Besides mere self-placement on the Likert scale parties were also asked to provide either a direct web link or a short textual justification for their self-placement positions. Responses were garnered from four political entities out of eleven, a figure consistent with historically observed modest party response rates in other Central European nations^29^. These self-placements were then calibrated against expert assessments. However, the final authority on party positioning rested solely with our Volebný Kompas team. The absence of a response from a party meant that its initial placement was determined exclusively by our expert coders. When placements by both coders and the party self-placement, if available, were in alignment, that position was automatically adopted as final. Any discrepancies were addressed through an intragroup deliberative calibration procedure^29^, ensuring a consensus-driven outcome that bolstered the robustness of our coding.

### Volebný Kompas implementation and usage

Volebný Kompas utilizes the euandi2019 platform designed for the 2019 European Parliamentary elections. The platform was originally developed by the Zurich-based company, xUpery Ltd., under the name “Societly”. The resilience and reliability of the euandi2019 platform are evidenced by its consistent implementation in a series of major electoral events, from the European Parliamentary elections in 2019 and the German federal elections in 2021, to the 2022 French presidential elections, the 2022 Italian parliamentary elections, and the Estonian parliamentary elections in 2023. This extensive and varied application across multiple electoral contexts underscores the platform’s tried-and-tested nature, highlighting its capability to consistently deliver in diverse settings^29,30,36^.

Upon interacting with Volebný Kompas, users were prompted to express their opinions on 39 statements using a five-point scale. An additional ‘no opinion’ option was provided, mirroring the scale used for coding party stances. Subsequently, users could choose to answer or skip additional questions. These included selecting up to three of the 39 statements they found most salient, indicating which of the 11 parties they considered voting for in the 2023 Slovak parliamentary elections, specifying parties they would never vote for, and providing basic demographic details such as gender, age, and education. The age and education details utilized categorical scales. Before seeing the results, users were given the option to voluntarily share their email addresses for use by the Volebný Kompas team in follow-up communication with exclusively academic purposes.

Upon finishing, the platform’s results displayed the percentage of alignment between the user and each party, ranging from 0% (no alignment) to 100% (full alignment). To quantify this, user responses were numerically translated as:

- Completely disagree = 0
- Tend to disagree = 25
- Neutral = 50
- Tend to agree = 75
- Completely agree = 100

**Fig. 2:**
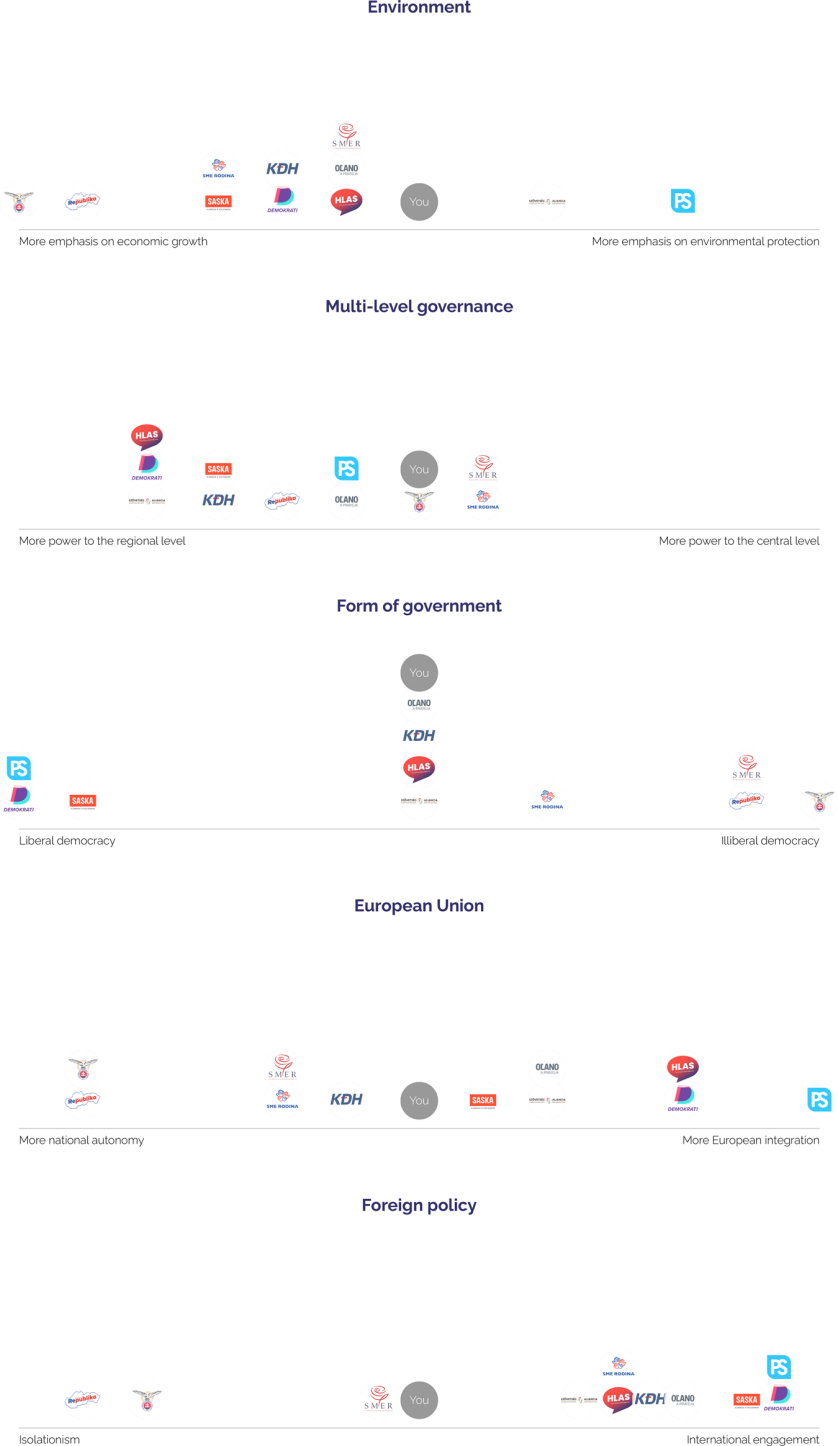
Slovak Political Parties’ Placement in Uni-Dimensions.

Political parties were assigned similar numerical values to represent their positions. The differences between user and party positions for each statement were calculated using the Manhattan distance. Besides displaying the closest parties based on users’ responses to the 39 statements, the platform also projected users’ and parties’ positions in five uni-dimensions (Fig. 2)^30^. These uni-dimensions correspond to more specific sub-dimensions of the political space that are particularly issue-relevant for a given electoral campaign, and are characterized by a general definition (e.g. ‘foreign policy’) and two opposing poles (‘isolationism’ vs. ‘international engagement’). Assuming that in most political systems individual opinions can be grouped into coherent categories, this tool shows users their proximity with parties in these specific categories, providing a useful cognitive shortcut for those who consider certain issues more important than others^27^. Out of the 39 statements, 18 were assigned to the five uni-dimensions (see Supplementary Table 1). Each of these 18 statements was given a polarity of either negative (−1) or positive (1). A positive polarity (1) increases the dimension score when users support a statement with a response within the range of 75–100. Conversely, a negative polarity (−1) increases the dimension score when users do not support a statement, indicated by a response within the range of 0–25. The resulting value for a political party or user ranges from 0 to 100.

**Fig. 3:**
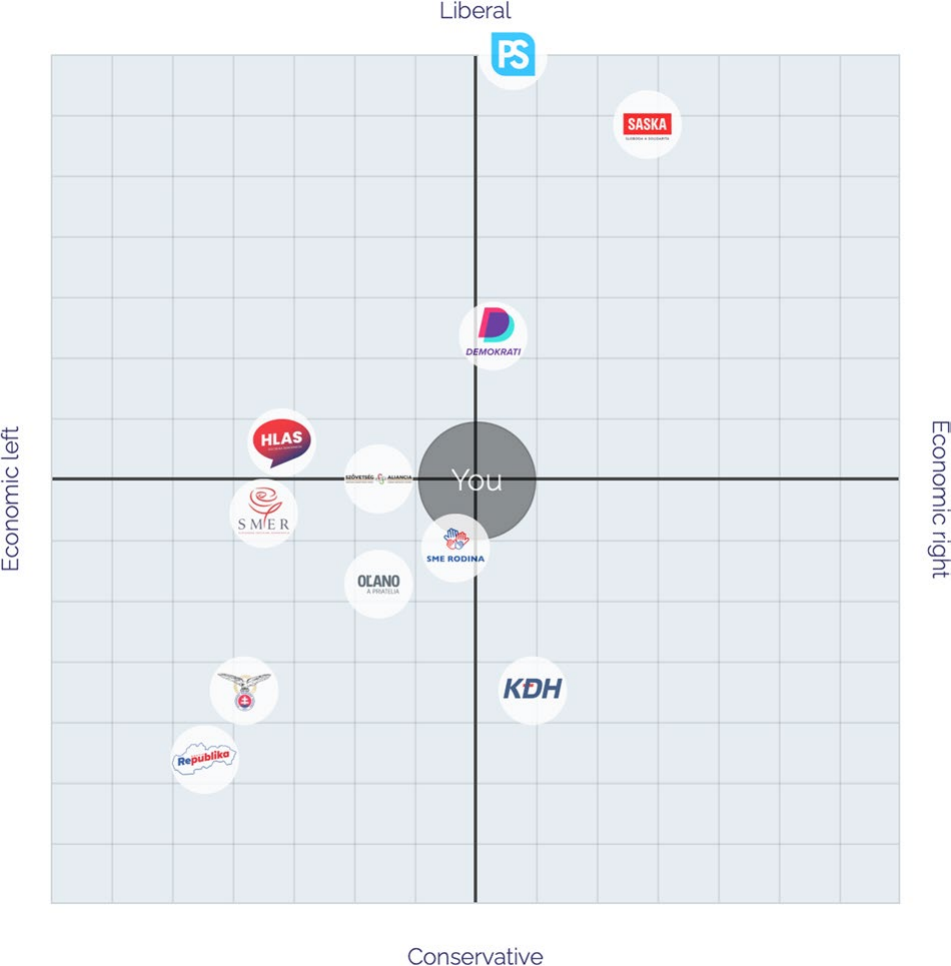
Slovak Political Parties’ Placement in Political Landscape.

To further simplify political alignment, the platform also placed users and parties on a political landscape represented in a two-dimensional space with socio-economic and socio-cultural axes, each ranging from 0 to 100 (Fig. 3). The initial position on both axes was set at 50 (neutral). The positions evolved based on responses to relevant statements, adjusted by the polarity values. Of the 39 statements, 16 influenced this two-dimensional political landscape. This representation reflects the established bi-dimensional structure of political cleavages in Europe^38^. Two independent coders assigned the dimensions and polarity statements. Any discrepancies between the coders were resolved through an intragroup deliberative calibration procedure, similar to the methodology used for party placements. The calculation for placements in both the uni-dimensions and the two-dimensional political landscape followed the euandi methodology^30^.

Volebný Kompas was officially launched and made publicly available on September 5th, 2023 (at https://euandi2019.eui.eu/survey/sk/slovensko2023). Given the nation’s political landscape, questions of representativeness were inevitable. To address these concerns and ensure a comprehensive capture of public sentiment, we adopted a multifaceted outreach strategy. While partnerships with leading media outlets like the Slovak news portal, DenníkN, and the national broadcaster, RTVS, were instrumental, we also recognized the need to penetrate deeper into the Slovak populace by also partnering with regional media. However, coverage was not limited to regional and national media alone; a significant digital wave on social media platforms also amplified Volebný Kompas’s visibility. High-profile political figures from both sides of the aisle, alongside ordinary citizens, actively re-shared and discussed their Volebný Kompas results, also leading to a proliferation of memes and online discussions centered on the platform. A testament to its widespread acceptance, President of Slovakia Zuzana Čaputová endorsed Volebný Kompas through videos on both Facebook and Instagram. The Instagram video alone attracted over 32,000 likes, with the President further emphasizing its importance by embedding a link to Volebný Kompas in her official Instagram bio.

**Fig. 4:**
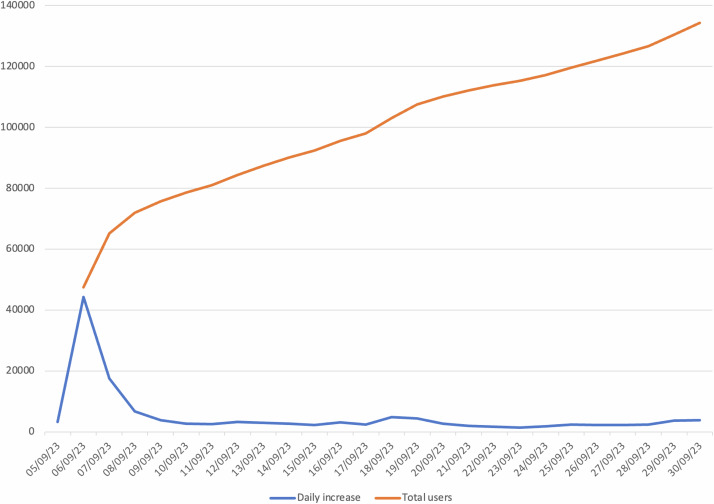
Volebný Kompas User Engagement.

The platform’s prominence, underscored by endorsements and media collaborations, is also supported by its high usage metrics, registering 270,836 website views within the 26-day span leading up to the elections. Figure 4 captures this momentum, showing “Complete” and user sessions and the daily increase.

## Data Records

The Volebný Kompas data are stored on the Figshare platform. The primary data files encompass the VAA user responses and coded political party positions. Each dataset is curated and structured for ease of analysis and interpretation.

**Repository Information**:

**Platform**: Figshare

**Access**: the datasets and affiliated files are publicly available at 10.6084/m9.figshare.24949470^39^.

**User Responses Dataset**:

**Format**: CSV

**Access**: data_without_email.csv

This raw dataset provides *n* = 134,699 individual Likert-scale responses to a series of 39 policy issues and value statements along with additional user demographics and preferences for most of the responses (i.e. age, education, gender, three most salient topics out of the 39 listed statements, parties each respondent would consider voting for, parties the respondent would never consider voting for, e-mail address). Each row corresponds to a unique response, with columns representing answers to various statements, voting preferences, and demographics.

**Party Positions Dataset**:

**Format**: Excel (xlsx)

**Access**: VAA_party_positions coded.xlsx

This dataset lists the *n* = 39 statements used in the survey across three languages — Slovak, Hungarian, and English and offers detailed information on various parties’ positions concerning specific statements with *n* = 429 observations. The data contains both party self-placements and expert-coded positions, further supported by position sources, snippets, and links.

To facilitate the analysis, researchers can readily import these datasets into any statistical software or platform supporting CSV and Excel formats. No additional proprietary software is required for accessing or analyzing the data. However, specific libraries or packages might enhance the data analysis process, especially when conducting specialized analyses or dealing with a larger dataset like the User Response Dataset. Along with the Volebný Kompas data, we also provide R code for pre-processing the data and conducting basic analysis. To illustrate the granularity and richness of the data, Table 1 provides an overview of columns from the ‘User Responses Dataset’, detailing the column name, description, and a sample entry. User responses in the ‘User Responses Dataset’ have been pseudo-anonymized, removing email addresses and replacing them with placeholder IDs so that individual responses remain anonymous, prioritizing user privacy.

**Table 1:** Overview of columns from the User Responses Dataset^*^.

Column Name	Description	Sample Entry
TIME.STAMP	Timestamp of the response	2023-10-09 08:57:38
S1	Likert scale response to statement 1	25.0
S2	Likert scale response to statement 2	75.0
…	…	…
UN26	Party that the user would not vote for (0: No / 1: Yes)	1
GENDER	User gender	Female
AGE	User age	21–24
EDUCATION	User education	Secondary education
EMAIL_ID	Pseudonymized email placeholder ID	ID E2

^*^Note: The “…” in the table above is used to represent continuation, as the full table is extensive.

A detailed data dictionary explaining the various variables in the datasets is also available in the associated figshare folder. This dictionary provides a comprehensive breakdown of each column, offering insights into its significance and context. In addition, the dictionary provides information on various other datasets concerning voter and party positions across multiple countries, which can be used for comparative purposes with the Volebný Kompas datasets.

The detailed structure of all associated files, along with the two primary datasets and R code, is documented in the README text available in the corresponding Figshare repository.

## Technical Validation

The validation process implemented for the Volebný Kompas datasets was aimed at ensuring the quality and consistency of the data. This process consisted of a series of systematic checks, both structural and content-based, to identify and rectify any discrepancies or anomalies within the data.

### Data cleaning

Prior to validation, the ‘User response Dataset’ underwent a data cleaning process. Responses that contained a ‘−1’ in the Likert scale columns (S1 to S39) were considered incomplete (i.e. users answering only a subset of the 39 statements) and thus removed. For the demographic variables ‘Age’, ‘Gender’, ‘Education’, and ‘Email ID’, empty strings were converted to **NA** to accurately represent instances where respondents elected not to answer. Similarly, empty strings in the Likert scale responses, representing a neutral position or ‘no opinion’, were re-coded to a value of 50. Otherwise, if excluded from the calculation, users answering only a few statements would be placed too close to certain parties. The raw, unprocessed data are available in the figshare repository; however, the R scripts provided alongside the dataset enable researchers to replicate the cleaning steps or modify them according to their research needs.

### Structural validation

After cleaning, datasets underwent structural validation to ensure data quality. This included univariate assessments for both main datasets and multivariate assessments specifically for the ‘User response Dataset’. These checks were carried out using the R package ‘validate’^40^. The ‘Party Positions Dataset’ being a small-N dataset was manually reviewed.

Univariate validation checks ensured that individual data points adhered to their expected formats and domains. For instance, fields such as ‘AGE’, ‘GENDER’, ‘EMAIL_ID’, and ‘TIME.STAMP’ were scrutinized for conformity to predefined categories and formats. Regarding the ‘EMAIL_ID’ entries, it was acknowledged that duplicates were permissible due to the potential for multiple submissions by the same respondent. Such allowances were factored into the validation logic.

Multivariate validation was conducted to ensure logical consistency across related variables. A notable aspect of this validation were the checks on alignment of ‘EDUCATION’ levels with corresponding ‘AGE’ categories. Upon initial review, we broadened the acceptable age range for those reporting a ‘University degree/Academic higher education’ to also include the ‘21–24’ category, which accounts for early graduates. We identified inconsistencies in the age-education relationship, specifically *n* = 40 cases under the age of 18 and *n* = 400 cases aged ‘18–20’, which were not congruent with university-level education attainment.

These discrepancies were acknowledged during further data curation and refinement of the data collection tool. The R scripts for these validation and cleaning procedures are available for review and use in the figshare repository.

#### Content validation

After the structural checks, content validation was conducted using statistical review and exploratory data analysis to ensure the dataset’s compliance with the expected quality standards for the study. This was in addition to the integrated checks and the application of reliable methodological approaches already described in the ‘Methods’ section of this article — such as calculating the weighted Cohen’s kappa for inter-rater reliability in statement coding, established party positioning methodology or utilizing a tried-and-tested online platform such as euandi2019 for response collection^29,30,36^.

Demographic distribution within the ‘User response Dataset’ indicates a skew towards younger, well-educated respondents. The pronounced presence of individuals with university degrees or academic higher education (*n* = 69,542, 64.41%) and the substantial numbers within the younger age brackets, particularly those aged 25–34 (*n* = 34,894, 32.32%) and 35–44 (*n* = 27,847, 25.79%), highlight this trend (Table 2). Despite this skewness, the extensive sample size of our dataset ensures a considerable representation across all age groups, including the older and less-educated demographics. For instance, while the younger age brackets, such as those aged 25–34 years, are prominently represented with *n* = 34,894 individuals, the dataset also includes significant participation from the senior population, with *n* = 2,716 individuals aged 65–74 years and *n* = 683 individuals 75 years or older. Similarly, alongside the *n* = 69,542 individuals with university degrees or academic higher education, the dataset encompasses *n* = 990 individuals with no education and *n* = 1,665 with only primary education, ensuring a diverse educational composition.

**Table 2:** Overview of columns from the User Responses Dataset.

Demographic Variable	Category	Frequency	Percentage (%)
Gender	Female	44,919	41.61
	Male	61,626	57.08
	Other / Prefer not to say	1,420	1.32
Age	Under 18	1,264	1.17
	18–20	7,020	6.50
	21–24	12,280	11.38
	25–34	34,894	32.32
	35–44	27,847	25.79
	45–54	15,178	14.06
	55–64	6,083	5.63
	65–74	2,716	2.52
	75 or older	683	0.63
Education	No education	990	0.92
	Primary education	1,665	1.54
	Secondary education	30,644	28.38
	Applied higher education	2,570	2.38
	Vocational education	2,554	2.37
	University degree/Academic higher education	69,542	64.41

The extensive sample size of the ‘User response Dataset’ ensures in total numbers substantial representation across all age groups. Even if some groups are underrepresented proportionally, the sheer volume of observations suggests that certain assumptions needed for valid post-adjustment remain applicable^41^. Researchers aiming to enhance the generalizability of their findings from this dataset might thus consider applying various statistical adjustments^42–44^. However, it is important to recognize that achieving external validity for the entire Slovak population may not be the necessary or intended objective for every analysis conducted with this data. We touch on the issues of external validity and also mention potential adjustment strategies that researchers might explore in the ‘Usage Notes’ section of the article.

**Fig. 5:**
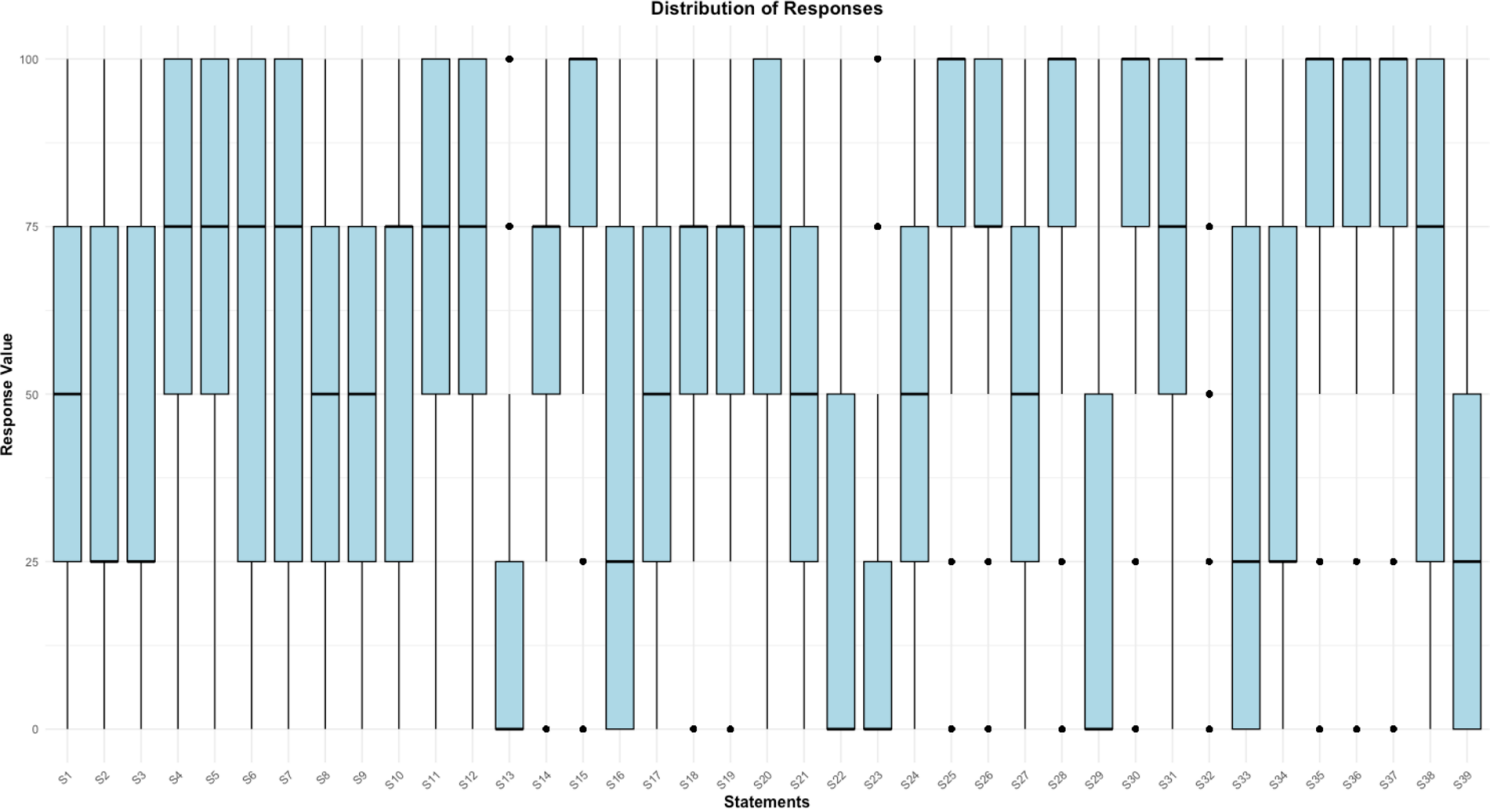
Distribution of responses for all statements.

In this part, we present computed summary statistics examples to illustrate the breadth of responses in the ‘User response Dataset’. The results indicate a wide range of participant opinions (Fig. 5), prior to the re-coding of ‘no opinion’ with a value of 50. Notably, statements such as S2 — ‘People who earn higher wages should have a higher income tax rate’ (*mean* = 43, *s.d*. = 33, median = 25) and S3— ‘The retirement age should rise as life expectancy increases’ (*mean* = 42, *s.d*. = 33, median = 25) highlight the diversity of opinions. Such variations are present across the dataset and indicate that multiple issues generate a broad spectrum of perspectives, suggesting that the dataset provides a solid snapshot of the population’s attitudes. Conversely, higher median scores on items such as S4 — ‘In the health care system, more emphasis should be placed on the personal responsibility of the individual’ (*mean* = 70, *s.d*. = 26, median = 75,) and S5 —‘Banks and big corporations should be taxed more heavily’ (*mean* = 69, *s.d*. = 29, median = 75) suggest a stronger consensus among respondents on these views. However, given the large sample size of the dataset, there are still substantial numbers — more than *n* = 17,435 for S4 and *n* = 23,344 for S5 — diverging on these views, with scores of 0 and 25. Our findings, particularly for questions that have been previously studied, are consistent with the expected socio-economic and political divisions within the Slovak populace, thereby lending further content validity to the dataset^4,15^.

**Fig. 6:**
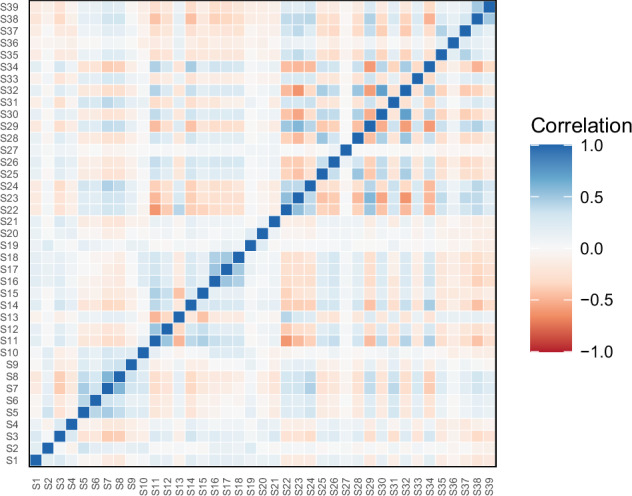
Pearson correlation matrix for responses to statements S1 to S39.

The ‘User response Dataset’ also underwent exploratory factor analysis (EFA) using R’s ‘psych’ package after additional pre-processing to ensure data integrity^45^. This pre-processing included reversing negatively worded statements and calculating both polychoric and Pearson correlation matrices independently^46^ (Fig. 6).

Both parallel analyses indicated that 11 factors should be extracted. To further account for potential model misspecification due to handling of Likert scale data, various estimation strategies were employed, including Maximum Likelihood (ML) and principal axis factoring, with promax rotation. This approach yielded consistent results. Notably statements such as S1 — ‘Real estate tax should be increased to lower labor taxes rises’ and S3 — ‘The retirement age should rise as life expectancy rises’ had strong loadings, representing distinct economic ideologies. The factors, delineated by items with high loadings, demonstrate a combination of shared and unique item variances, capturing a diverse spectrum of opinions within the dataset. The factor structure largely aligns with the expected socio-economic and political constructs. Nevertheless, the complexity of the factors identified necessitates further examination. The EFA’s findings seem to propose a valid measure of socio-political attitudes; however, we recommend additional validation, including confirmatory factor analysis (CFA) and expert review. While EFA provides a statistical basis, relying solely on voter responses for interpretation should be avoided, emphasizing the need for theoretical support and potentially also for alternative methods such as Mokken Scaling Analysis (MSA) for further refinement^47^.

**Fig. 7:**
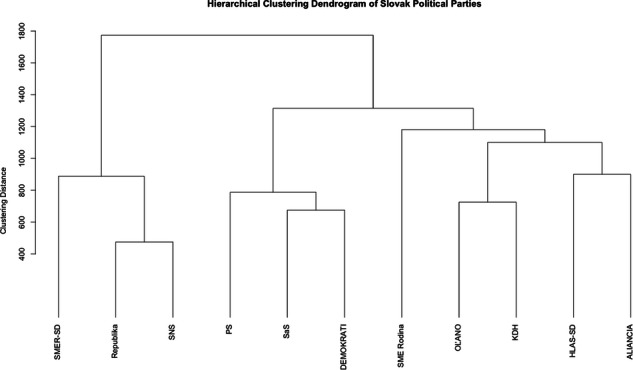
Hierarchical Clustering Dendrogram of Slovak Political Parties.

On the supply side of Slovak politics, given the naturally limited number of observations in the ‘Party Positions Dataset’, factor analysis proved unsuitable due to an unstable and non-invertible covariance matrix. Instead, hierarchical clustering was employed to delineate how political parties group based on their policy stances, thereby identifying underlying dimensions within the Slovak political space. We used the ‘recoded_party_positions’ dataset and computed pairwise Manhattan distances to emphasize the absolute differences in policy positions, applying average linkage clustering to identify groups of parties based on policy similarity. The clustering results were visualized in a dendrogram (Fig. 7). The results largely conform to the anticipated clustering of Slovak parties, thereby bolstering the validity of the dataset. However, despite its benefits, this approach has certain limitations, as it might yield different results based on, inter alia, linkage criteria and distance measures, leading to varied interpretations. For further research on this we thus recommend comparing different clustering techniques in conjunction with expert review to ensure robustness.

For an additional validity check on the ‘Party Positions Dataset’, we also manually reviewed the results of the five uni-dimensional measures and political landscape dimensions (Figs. 2 and 3) produced by our Voting Advice Application (VAA) platform. The results conform to anticipated placements of Slovak parties within the political space as well as to results of the hierarchical clustering dendrogram (Fig. 7)^48^, thus further lending validity to the dataset.

## Usage Notes

When using the Volebný Kompas ‘User Response Dataset’, it is crucial to consider the specific analytical objectives one aims to achieve, given that attaining external validity for the entire Slovak population may not always be the desired or appropriate goal for every analysis utilizing the data. Preliminary evaluations of the dataset’s demographics indicate a positive skewness on average toward younger, well-educated respondents. If ones goal is to enhance the generalizability of findings from this dataset, researchers might consider using statistical adjustments such as post-stratification, raking, or multilevel regression with post-stratification (MRP)^43^. MRP, in particular, has been shown in recent years to generate accurate survey estimates from non-representative samples, especially with larger sample sizes^44^. These techniques might help calibrate the sample to more closely reflect the characteristics of the target population, provided certain assumptions hold^41^. To define the coefficients necessary for these approaches, we provide links to the Slovak census dataset as well as an already pre-processed CSV file containing the required demographic variable distributions from the Slovak census data, all of which are available in the Figshare repository. Besides descriptive inference, researchers interested in causal inference can also utilize the data. Although there are various challenges associated with causal inference from observational data, employing methodological approaches^49^ — such as nonparametric pre-processing with matching followed by estimation^50^, or using more sophisticated techniques like Structural Causal Models (SCMs)^51^ — may yield accurate estimates. Nonetheless, even when employing the aforementioned methods, researchers must exercise caution regarding their estimates. The necessity for state-of-the-art knowledge about the phenomena under study is paramount, and the importance of conducting robustness checks and sensitivity analyses cannot be overstated^51,52^. The Volebný Kompas data can however also serve as an integral source of evidence that can be utilized alongside other datasets in mixed-methods analyses, offering resources for both comparative and longitudinal studies focused on political behaviour in the region. The datasets also allow analysists to link complex array of correlations in Fig. 6 with respondents’ expressions of what parties they would and would not vote for and with respondents’ choices of their three most salient issues. Together this array of data opens up a wide variety of possibilities: finding clusters among issues that allow the mapping of a multi-dimensional issue space, connecting issue positions with party preference (and opposition) to map the *partisan* issue space, linking overall issue preferences and preferences of party supporters that allows the calculation of issue valence and introduction of salience measures into each of these calculations to account for the role of issue importance^53^. Furthermore, the datasets are an invaluable asset for researchers interested in studying Voter Advice Applications (VAAs), providing a wealth of information for those examining the evolution and impact of such tools.

## Supplementary information

Supplementary Table S1

## Data Availability

The Volebný Kompas datasets are publicly available at 10.6084/m9.figshare.24949470^39^. Code to replicate the main analyses in this paper is publicly available at 10.6084/m9.figshare.24949470^39^.
